# Pan-genome analyses identify lineage- and niche-specific markers of evolution and adaptation in *Epsilonproteobacteria*

**DOI:** 10.3389/fmicb.2014.00110

**Published:** 2014-03-19

**Authors:** Ying Zhang, Stefan M. Sievert

**Affiliations:** Biology Department, Woods Hole Oceanographic InstitutionWoods Hole, MA, USA

**Keywords:** pan-genome, core genes, flexible genes, *Epsilonproteobacteria*, *Sulfurimonas*, *Helicobacter*, *Campylobacter*

## Abstract

The rapidly increasing availability of complete bacterial genomes has created new opportunities for reconstructing bacterial evolution, but it has also highlighted the difficulty to fully understand the genomic and functional variations occurring among different lineages. Using the class *Epsilonproteobacteria* as a case study, we investigated the composition, flexibility, and function of its pan-genomes. Models were constructed to extrapolate the expansion of pan-genomes at three different taxonomic levels. The results show that, for *Epsilonproteobacteria* the seemingly large genome variations among strains of the same species are less noticeable when compared with groups at higher taxonomic ranks, indicating that genome stability is imposed by the potential existence of taxonomic boundaries. The analyses of pan-genomes has also defined a set of universally conserved core genes, based on which a phylogenetic tree was constructed to confirm that thermophilic species from deep-sea hydrothermal vents represent the most ancient lineages of *Epsilonproteobacteria*. Moreover, by comparing the flexible genome of a chemoautotrophic deep-sea vent species to (1) genomes of species belonging to the same genus, but inhabiting different environments, and (2) genomes of other vent species, but belonging to different genera, we were able to delineate the relative importance of lineage-specific versus niche-specific genes. This result not only emphasizes the overall importance of phylogenetic proximity in shaping the variable part of the genome, but also highlights the adaptive functions of niche-specific genes. Overall, by modeling the expansion of pan-genomes and analyzing core and flexible genes, this study provides snapshots on how the complex processes of gene acquisition, conservation, and removal affect the evolution of different species, and contribute to the metabolic diversity and versatility of *Epsilonproteobacteria*.

## Introduction

The evolution of bacterial genomes is characterized by a massive amount of insertions, deletions, and rearrangements, permitting the differentiation and adaptation of evolutionarily related lineages into vastly diverse environments (Romero and Palacios, 1997; Cohan, 2001; Mira et al., 2002; Reams and Neidle, 2003). Over the past two decades, the realization of extended pan-genomes in bacterial species has stimulated many discussions about the meaning of species boundaries (Medini et al., 2005). The fact that strains within a single species can have widely different repertoires of genes has highlighted the complexity in identifying bacterial species. Despite the complete sequencing of over 2000 bacterial genomes and the in-depth study of a number of model organisms, a coherent model is still lacking for genome evolution both within and among bacterial species.

In practice, bacteria have been classified using a polyphasic approach combining information from multiple molecular, morphological, and physiological analyses (Rosselló-Mora and Amann, 2001), with molecular methods playing an increasingly important role. The universally conserved 16S ribosomal RNA (rRNA) gene has been widely used to assess the phylogenetic diversity and, by inference, even the functional diversity of microbes in environmental samples. However, inferring function based on the 16S rRNA gene is only possible in selected cases where all members of a 16S-defined clade share the same physiology, e.g., in case of cyanobacteria or certain groups of sulfate-reducing *Deltaproteobacteria*. Further, various studies have demonstrated substantial genomic variations among strains that differ only slightly in 16S rRNA sequences (Coleman et al., 2006; Rasko et al., 2008; Tettelin et al., 2008). Therefore, a genome-scale understanding of how genes evolve and what determines the acquisition and deletion of genes is essential for mapping the complete genetic variations of bacteria, while at the same time assisting in the classification and functional identification of bacterial species.

Traditionally, the term pan-genome has been used to describe the full repertoire of genes found in different strains of a single species (Hanage et al., 2005; Konstantinidis et al., 2006; Read and Ussery, 2006; Lefébure et al., 2010; Lukjancenko et al., 2010), but more recently this concept has been extended to represent the total genes in any pre-defined group of bacteria or archaea (Polz et al., 2013). Here, we compared the pan-genomes at different taxonomic ranks within the class *Epsilonproteobacteria*. By examining the genomic variations within same species as well as among different species, we investigated how the processes of gene conservation and transfer affect the evolution of pan-genomes and contribute to the functional adaptation of individual species.

The class *Epsilonproteobacteria* is metabolically diverse and contains organisms with different life styles, including both free-living and host-associated species (Campbell et al., 2006). To date, most studies have focused on species that are associated with the human digestive system, such as members of the genera *Helicobacter* and *Campylobacter*, where they exist either asymptomatically or cause diseases like peptic ulcers or gastric cancer (Engberg et al., 2000). The environmental relevance of *Epsilonproteobacteria* had not been recognized until the late 90′ s (Moyer et al., 1995; Polz and Cavanaugh, 1995; Longnecker and Reysenbach, 2001). As more and more free-living species were identified and isolated, it became clear that *Epsilonproteobacteria* play important roles in the biogeochemical cycling of nitrogen, sulfur, and carbon in various marine and terrestrial environments (Campbell et al., 2006). At deep-sea hydrothermal vents, chemoautotrophic *Epsilonproteobacteria* serve as important primary producers by utilizing the abundantly available geochemical energy sources to assimilate inorganic carbon through a process known as chemosynthesis (Sievert and Vetriani, 2012, and referenes therein). *Epsilonproteobacteria* also play important roles in coastal and open ocean environments characterized by reducing conditions, such as sulfidic sediments, euxinic water columns, and oxygen minimum zones (e.g., Sievert et al., 2008; Labrenz et al., 2013).

The present study aimed at understanding the function and evolution of the pan-genomes of *Epsilonproteobacteria* at three different levels of taxonomy: species, genus, and class. To this end, we compared all published full genomes of *Epsilonproteobacteria* at the time of our study (Table S1). These genomes represented a wide-ranging set of isolates and provided an excellent opportunity for studying the connections between genome diversity and phenotypic diversity. Specifically, we set out to answer three fundamental questions. First, do the pan-genomes of different taxonomic ranks within *Epsilonproteobacteria* show different rates of expansion? Second, how is the evolution of the class reflected in the core genes of its pan-genome? Finally, how are the adaptive features reflected in flexible genes of a species?

## Results

### Modeling the expansion of *Epsilonproteobacterial* pan-genomes

The expansion of a pan-genome can be examined by plotting the number of genomes considered against the total number of genes observed. The associations on the plot can then be mathematically evaluated through fitting regression models (Tettelin et al., 2008), according to which it can be classified as “open” or “closed” (see Materials and Methods for details). While the size of an open pan-genome would increase unboundedly with the inclusion of new genomes, the size of a closed pan-genome would reach a plateau after a certain number of sample genomes were included. Previously, such analyses have only been performed at the species level (Tettelin et al., 2008). In order to compare the pan-genomes of different taxonomic ranks, we constructed regression models for five groups of *Epsilonproteobacteria* that correspond to three different levels of taxonomy (Table S1): two groups representing strains of same species (intra-species), two representing species of same genera (intra-genus), and one representing different species of the entire class (intra-class or inter-species). It is worth mentioning that groups of higher taxonomic rank encompassed those of lower rank. For example, the inter-species group (Class) included all the different species in the intra-genus groups (Genus), and the intra-genus groups included a representative strain from each of the intra-species groups (Species). Hence, our analyses compared the extent of pan-genome expansion at all three taxonomic levels.

**Figure 1:**
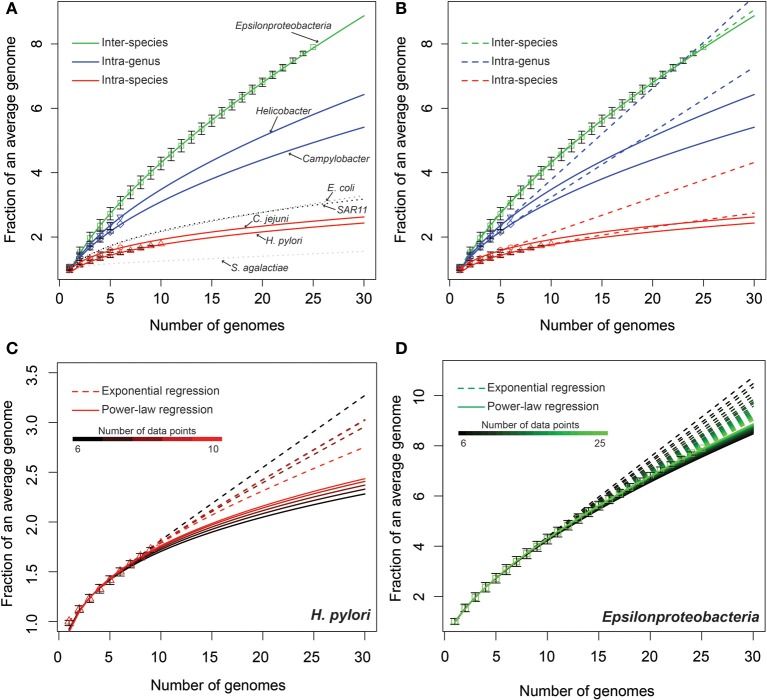
**Modeling epsilonproteobacterial pan-genomes under different taxonomic levels.** The curves show the extrapolated models. The data points present the median size of pan-genomes over complete or random permutations (Materials and Methods), normalized by the median sizes of individual genomes in respective genome sets, and the error bars represented the 25th and 75th percentiles (Materials and Methods). **(A)** Power law models. The curves for *E. coli* (Rasko et al., 2008), *Sar11* (Grote et al., 2012), and *S. agalactiae* (Tettelin et al., 2005) were plotted following published data. **(B)** Comparison of power law models (solid curves) with exponential models (dotted curves). **(C,D)** Evaluating the stability of power-law versus exponential models by varying the number of genomes (data points) included in the extrapolation.

We used a two-step process to model the expansion of the pan-genomes. First, complete or random permutations were carried out with step-wise additions of new genomes, and a median was taken on the size of pan-genomes after each step. Second, the median counts was extrapolated using two different models: power-law regression (Tettelin et al., 2008) and exponential regression (Tettelin et al., 2005). The resulting extrapolations were normalized by the median genome sizes in their respective sets to assist the visualization and comparison of the fitted curves (Figure 1).

The power-law regression showed that all five pan-genomes in our dataset are open, with an average γ parameter of 0.29, 0.53, and 0.66, respectively, for intra-species, intra-genus, and inter-species sets (Materials and Methods). The extrapolated curves of intra-species (red), intra-genus (blue), and inter-species (green) pan-genomes followed distinct slopes: while the intra-species curves were the shallowest, the inter-species curves were the steepest. In contrast, the curves within the same category (the two intra-species or the two intra-genus groups) presented similar slopes (Figure 1A and Table S2). While both intra-species curves (*H. pylori* and *C. jejuni*) were slightly shallower than the pan-genome curves of *E. coli* (Rasko et al., 2008) and SAR11 (Grote et al., 2012) and steeper than that of *Streptococcus agalactiae* (Tettelin et al., 2005), the two intra-genus curves (*Helicobacter* and *Campylobacter*) were much steeper than all evaluated intra-species curves (Figure 1A).

The power-law model fit the available data with high *R*^2^ values of more than 0.98 for all extrapolations, while the exponential model had on average lower *R*^2^ values, especially when the number of considered genomes is relatively small (Table S2). To further evaluate this, we fitted both models to a varying number of data points to monitor how the number of available genomes may affect the accuracy of pan-genome modeling (Figures 1C,D). For example, when using a range of 6–10 considered genomes in the modeling of *H. pylori* (Figure 1C), the exponential curves (dotted lines) are more spread out than the power-law curves (solid lines). Similarly, modeling of the entire *Epsilonproteobacteria* also showed that the power-law model is more stable than the exponential regression model and less influenced by the availability of genomic data (Figure 1D). Besides this, results in Figures 1C,D also showed that the number of available genomes are far from saturating the power-law model. In other words, new genes are still been discovered with the addition of each genome. Therefore, the diversity of epsilonproteobacterial pan-genomes is yet to be fully explored with additional genomic sequences.

### Classifying the core, flexible, and singleton genes in the pan-genome

The pan-genome of all the examined *Epsilonproteobacteria* contains 16,349 clusters of non-redundant protein coding genes (Materials and Methods). Among them, 289 clusters (1.7%) represented Conserved Single Copy Genes (CSCGs) that appear only once in every examined genome. These CSCG clusters contain 11,271 genes, which account for 16% of the more than 70,000 genes in all analyzed genomes. At the other end of the spectrum are 10,944 clusters (67%) that occur only in a single genome, accounting for about 15% of the total genes. We classified the total genes in the pan-genomes into three sets based on their occurrence: (1) the “core” genes, which are universally conserved in all considered genomes, include both CSCGs and conserved genes of multiple copies per genome, (2) the “singleton” genes are specific to single genomes, and (3) the “flexible” genes are found in more than one, but not all genomes.

**Figure 2:**
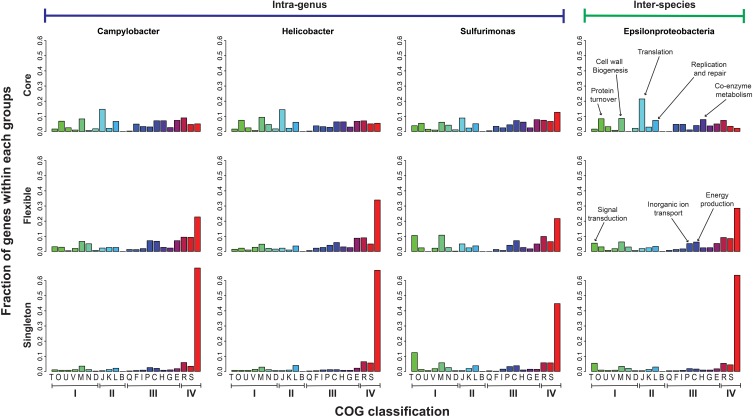
**Functional distribution of the Core, Flexible, and Singleton genes in the pan-genomes of the class *Epsilonproteobacteria* and the genera of *Campylobacter*, *Helicobacter*, and *Sulfurimonas*.** The X-axis was labeled with the one-letter codes that correspond to different functional categories in the COG database (Tatusov et al., 2003), which was further grouped based on the broader categories: I—Cellular processes and signaling, II—Information storage and processing, III—Metabolism, and IV—Poorly characterized or unmapped. The right-most bars were unlabeled and indicate the “unmapped” category. The Y-axis indicates the fraction of genes in a COG family out of the total genes in each of the core, flexible and singleton groups.

The functional distribution of core, flexible, and singleton genes was examined for the intra-genus groups of *Helicobacter*, *Campylobacter*, and *Sulfurimonas*, as well as the inter-species group. Figure 2 shows the classifications based on the Clusters of Orthologous Groups (COG) database (Tatusov et al., 2003). The genes that cannot be classified into any existing COG clusters were grouped into the “unmapped” category. For all groups, the majority of the singleton genes (56–79%) and a large fraction of the flexible genes (38–48%) were unmapped or poorly characterized, while only a small fraction of the core genes (13–27%) had no clear functional assignment. The core, which represents an indispensable part of all genes in *Epsilonproteobacteria*, contained mainly housekeeping genes that encode the central machinery of a cell, such as translation, protein modification and turnover, replication and repair, cell wall biogenesis, as well as co-enzyme metabolism. In contrast, the flexible and singleton genes that could be mapped to a functional category mainly encoded functions in signal transduction, inorganic ion transport, and energy production.

While both considered to be non-core (Medini et al., 2005), the flexible and singleton genes presented slightly different functional distributions. A larger fraction of the flexible genes (66–78%) were mapped to existing COG families than the singleton genes (33–55%) despite both being poorly characterized. Additionally, a slightly larger fraction of the flexible genes (10–14%) encoded functions in energy production and inorganic ion transport than the core (5–12%) and singleton (3–7%) genes. Therefore, these processes were more likely shared among subgroups of *Epsilonproteobacteria* than being common to all or unique to a single species. In the following two sections, we further investigate the composition and evolution of pan-genomes by analyzing the core and flexible genes. First, phylogeny of the core genes was reconstructed and used to infer the evolutionary history of *Epsilonproteobacteria*. Second, an analysis was carried out to identify lineage-specific and niche-specific signals within the flexible genome of selected species.

### Phylogenomic reconstruction based on core genes

**Figure 3:**
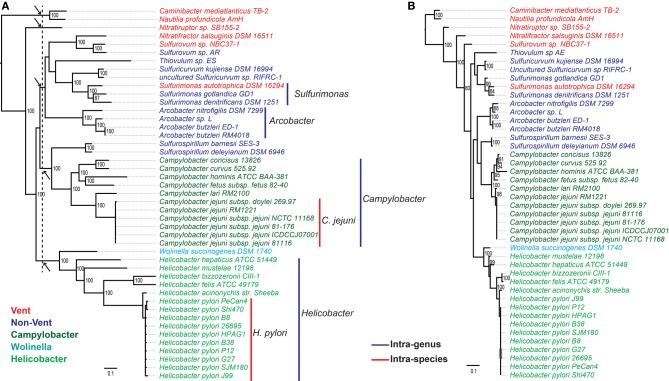
**Phylogeny of *Epsilonproteobacteria* based on (A) concatenated protein sequences of a subset of the CSCGs (Materials and Methods), and (B) 16S rRNA gene sequences.** The species labels are color-coded to reflect the taxonomic information and environmental conditions of the isolates. Specifically, the free-living species are classified into two groups, Vent (red) versus Non-Vent (blue), depending on whether or not a species was isolated from deep-sea hydrothermal vents. Whereas the host-associated species are classified into three groups, *Helicobacter* (green), *Campylobacter* (dark green), and *Wolinella* (cyan), in accordance with their taxonomic classifications. The phylogenomic tree in **(A)** is marked with colored vertical lines at the right-hand side to highlight taxonomic groups at the species (red lines) and genera (blue lines) levels. The dotted line that crosses the internal branches of **(A)** indicates the proposed cutoff for family-level taxonomic classification of *Epsilonproteobacteria*, and the roots of the five proposed families are identified with arrows.

**Figure 4:**
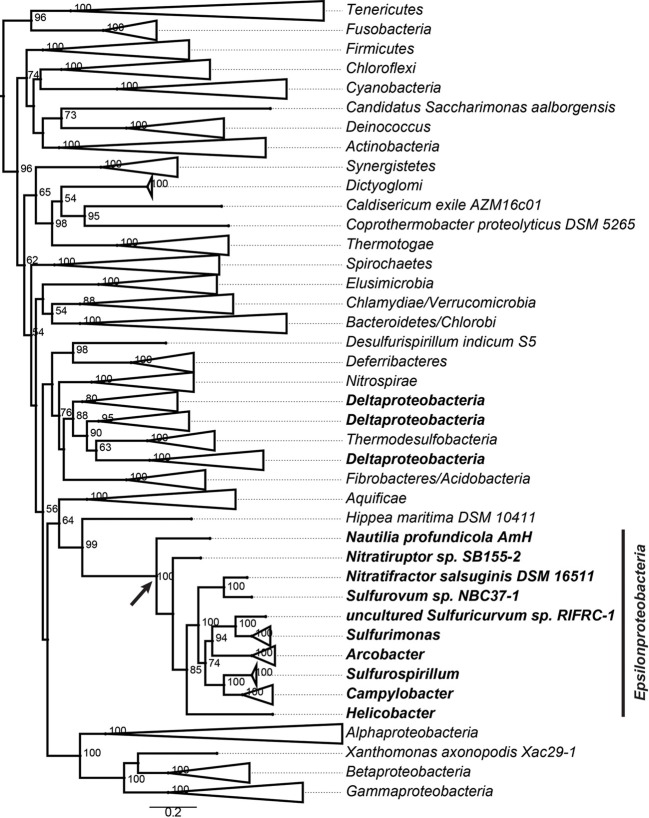
**Phylogenomic reconstruction of a global bacterial tree based on the concatenated protein sequences of 37 markers.** The leaves are collapsed when possible to present taxonomic groups. Bootstrapping values of more than 50% are shown. The *Deltaproteobacteria* is highlighted with bold font. The *Epsilonproteobacteria* clade is expanded to show individual species and genera, and the root of *Epsilonproteobacteria* is indicated with a black arrow.

The core genome of *Epsilonproteobacteria* contains 289 CSCGs and 40 conserved genes with multiple copies per genome. Specifically, the CSCGs accounted for 15% of all the genes in an average epsilonproteobacterial genome. The fact that these genes are universally present in a single copy makes them useful markers for inferring the phylogenetic relationships within the class (Figure 3A), as well as evaluating the phylogenetic position of the *Epsilonproteobacteria* as a whole (Figure 4).

The phylogenomic analysis (Figure 3A) is overall consistent with a phylogenetic tree based on 16S rRNA sequences (Figure 3B), in which species of the same taxonomic groups were tightly clustered. According to both trees, the deepest lineages within the *Epsilonproteobacteria* are represented by *Nautilia profundicola* (Smith et al., 2008) and *Caminibacter mediatlanticus* (Voordeckers et al., 2005), both of which are moderate thermophiles and obligate anaerobes isolated from deep-sea hydrothermal vents. The other species from either vent or non-vent environments emerged later in evolution, which paralleled the emergence of host-associated species. Despite these similarities, the CSCG-based tree (Figure 3A) was overall better resolved compared to the 16S rRNA gene tree (Figure 3B), and it indicated slightly different branching patterns of certain clades, such as the precise phylogenetic positions of the genera *Arcobacter* and *Helicobacter*.

Besides assisting in the evolutionary reconstruction within the *Epsilonproteobacteria*, the CSCGs can also help in evaluating the phylogenetic affiliation of this class as a whole (Figure 4). We used a set of 37 phylogenomic markers that are universally present in single copies in a set of fully sequenced genomes to build the bacterial tree. These markers included 31 universal genes published in a previous study (Wu and Eisen, 2008), as well as six additional genes that were identified through a global search of epsilonproteobacterial CSCGs using Hidden Markov Models (HMMs). The concatenated protein tree revealed a close affiliation of the *Epsilonproteobacteria* with the *Aquificae*, as well as provided evidence that the *Epsilonproteobacteria* represent a distinct clade that is separated from *Proteobacteria*. The global analyses also positioned *Deltaproteobacteria* into a distal branch. With the exception of *Hippea maritima* (Anderson et al., 2011), all other examined deltaproteobacterial genomes grouped with the phyla *Thermodesulfobacteria* (Anderson et al., 2012; Elkins et al., 2013), *Acidobacteria* (Ward et al., 2009; Challacombe et al., 2011; Rawat et al., 2012), and *Nitrospirae* (Lücker et al., 2010; Fujimura et al., 2012).

### Niche-specific genes at the deep-sea hydrothermal vents

While a subset of the core genes supported the phylogenomic reconstruction of *Epsilonproteobacteria*, they only covered a small fraction of an average genome. The remaining genes (85%) were either shared among a subset (flexible genes), or they only occurred in one genome (singleton genes) of currently sequenced *Epsilonproteobacteria*. Therefore, analyses of these genes can provide a more complete picture of evolution of this class. Since the singleton genes were largely unmapped to COG families (Figure 2) and because the definition of singleton genes is subject to change, i.e., what appears to be a singleton today may be reclassified into a multi-gene cluster when new genome data becomes available in the future, we decided to focus our study on the flexible genes.

**Figure 5:**
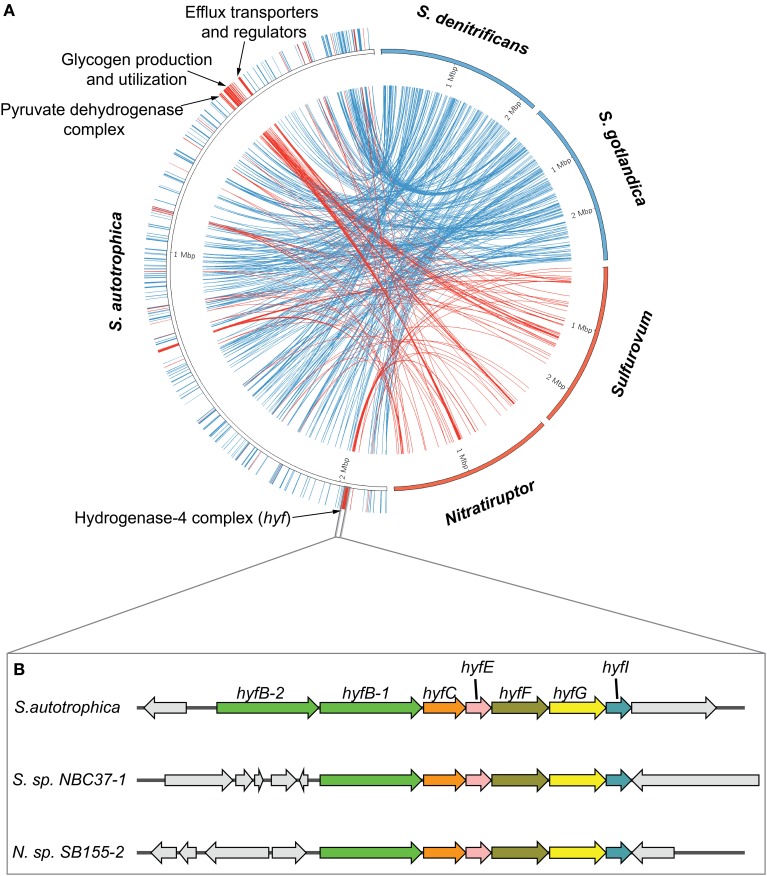
**Lineage-specific and niche-specific genes in *Sulfurimonas autotrophica*. (A)** Mapping of the *Sulfurimonas*-specific and vent-specific genes. The genomic sequence of *S. autotrophica* is represented by the left semicircle. The other four genomes, colored according to their groups (blue for *Sulfurimonas* and red for vent), were shown on the right, with each genome scaled to be 25% of the semicircle. The links in the center connect the genes that are uniquely shared among *Sulfurimonas* (blue) or vent species (red). **(B)** Cartoon diagram of a vent-specific operon with a putative function of a Formate hydrogenlyase complex based on annotations in the SEED database (Overbeek et al., 2005). The homologs were indicated by arrows with same colors and labeled with gene names.

We hypothesized that the flexible genome carries two types of information: (1) lineage specific signals that trace the evolutionary heritage of a single lineage, and (2) niche specific signals that represent the adaptation of different species to similar environments. We tested this hypothesis by comparing the genome of a free-living species from a particular environment to genomes of species within the same genus, but inhabiting different environments, as well as to genomes of other species inhabiting a similar environment, but belonging to different genera. We chose *Sulfurimonas autotrophica* for our studies, which was the only free-living species that matched our criteria. We compared the genome of *S. autotrophica* with two subsets (Figure 5), one containing its phylogenetic neighbors of the same genus that were isolated from different environments (*S. gotlandica*, and *S. denitrificans*), and the other containing organisms with similar habitats, but belonging to different genera (*Sulfurovum* sp. NBC37-1, and *Nitratiruptor* sp. SB155-2) (Figure 3).

Our analyses successfully identified genes that were either uniquely shared among the *Sulfurimonas* species, i.e., genus specific, or among the vent isolates, i.e., habitat specific (Figure 5). This confirmed our hypothesis that both lineage-specific and niche-specific evolutionary events were recorded in the flexible genome. Overall, there were almost three times as many genes specifically inherited within the genus of *Sulfurimonas* (195 unique genes, Table S3) than shared among the vent isolates (67 unique genes, Table S4). Further, the *Sulfurimonas*-specific genes were distributed evenly across the genome of *S. autotrophica*, while the vent-specific genes clustered tightly at certain locations of the genome. The vent-specific gene clusters encode three major functions, including glycogen production and utilization, inorganic ion and efflux transport, and energy production and conversion. We specifically focused on two vent-specific protein complexes related to energy production and conversion (Hyf-like and PYDH complex) as examples to further elucidate the evolution of vent-specific genes in the flexible genome.

**Figure 6:**
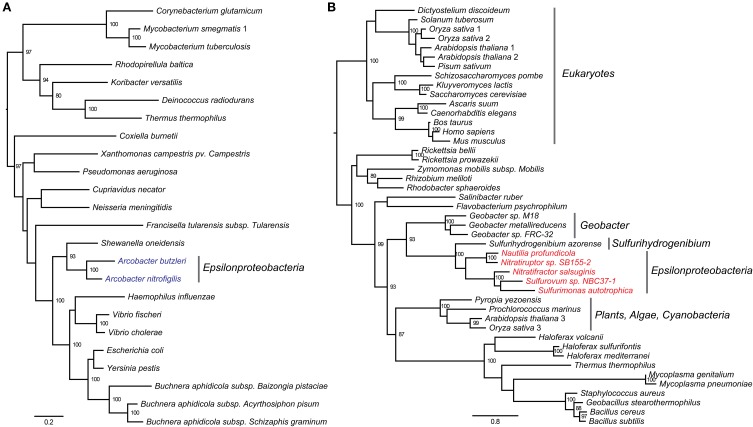
**Protein phylogeny of the type-I (A) and type-II (B) E1p enzymes of the pyruvate dehydrogenase complex.** The leaves of the tree were labeled with the genome names, and the internal nodes were labeled with bootstrapping values, in which only values of at least 80 were shown. **(A)** The type-I form of *Epsilonproteobacteria* was found within the genus *Arcobacter* (blue). **(B)** Alpha-subunit of the type-II form was used to construct the tree. The type-II form of *Epsilonproteobacteria* was only found in species isolated from deep-sea hydrothermal vents (red).

The vent-specific *hyf*-like operon (*hyfBCEFGI*) encodes a putative hydrogenase-4 complex (Figure 5B). This operon is conserved among all the vent species, while the genomic neighborhood of the operon is completely unrelated between different species (Figure 5B). Besides the *hyf*-like operon, all examined genomes of vent-inhabiting *Epsilonproteobacteria* also encode a homolog of formate dehydrogenase H (Fdh-H). This combination resembles the formate hydrogenlyase (FHL-2) complex of *E. coli*, which oxidizes formic acid to carbon dioxide and molecular hydrogen (Andrews et al., 1997). The pyruvate dehydrogenase complex (PYDH) is a multi-enzyme complex composed of three different enzymes: a decarboxylase (E1p), a dihydrolipoamide acyltransferase (E2p), and a dihydrolipoamide dehydrogenase (LPD), among which the E1p carries out the initial step of an enzymatic reaction that converts pyruvate to acetyl-CoA, NADH and CO_2_ (Neveling et al., 1998). The E1p can be present in two forms, one containing multiple copies of a single subunit (type-I) and the other containing multiple copies of two subunits, alpha and beta (type-II). These two forms are not evolutionarily related, and either or both forms may be present in the same organism (Schreiner et al., 2005). Our analyses confirmed the presence of PYDH in seven epsilonproteobacterial genomes: two encode the type-I form and belong to the genus *Arcobacter*, while the other five encode the type-II form and are from species inhabiting deep-sea hydrothermal vents (Table S5). We constructed phylogenetic trees of the E1p enzymes in order to further investigate the evolution of the two forms of PYDH in *Epsilonproteobacteria* (Figure 6). According to the phylogenetic trees, the type-I form, which existed solely in the genus *Arcobacter*, was closely related to the PYDHs encoded in *Gammaproteobacteria* (Figure 6A), whereas the type-II form, which existed solely in the vent species, grouped with the enzymes of *Sulfurihydrogenibium azorense*, which belongs to the *Aquificales*, and of *Geobacter* species (Figure 6B).

## Discussion

The recognition of bacterial pan-genomes has resulted in discussions regarding the bacterial species concept (Hanage et al., 2005; Konstantinidis et al., 2006; Read and Ussery, 2006; Lefébure et al., 2010; Lukjancenko et al., 2010). A fundamental problem in microbiology today remains how to define bacterial species according to their genomic information, or in other words, how to accurately reconstruct the acquisition, removal, or conservation of genes during divergence and speciation. Here, we approached this problem using a case study of the class *Epsilonproteobacteria* by systematically comparing all available genomes from this class. We quantitatively modeled the expansion of pan-genomes at the intra-species, intra-genus, and inter-species levels using current taxonomic classifications. Our model not only indicates a steady rate of conservation and divergence for epsilonproteobacterial genomes of the same taxonomic rank, but also verified that strains of a same species have much lower levels of genomic variation than the different species (Figure 1). Moreover, comparing two different approaches of modeling bacterial pan-genomes, our study suggested the power-law regression provides a better fit and is more stable than the approach based on the exponential regression, especially when the number of available genomes is low (Figures 1C,D).

While the modeling of bacterial pan-genomes can be useful for evaluating the overall genomic diversity, the analyses of individual or subgroups of genes can provide a detailed picture of genomic and functional evolution. Typically, a pan-genome is composed of a core and a non-core fraction, and the phylogeny of CSCGs in the core can provide important insights into the evolution of various lineages. We performed evolutionary reconstruction using the concatenated protein sequences encoded by a subset of CSCGs and compared it with a reconstruction based on the 16S rRNA genes (Figure 3). In general, both approaches agreed with each other, further corroborating that *Epsilonproteobacteria* evolved from thermophilic and anaerobic species, and subsequently diversified into mesophilic and microaerobic species that colonized non-vent environments, as well as became host-associated mutualists, commensals or pathogens (Campbell et al., 2006). Compared to the current taxonomic classification that features two main families, i.e., *Campylobacteraceae* and *Helicobacteraceae*, the CSCG-bsed phylogenomic reconstruction suggests a new scheme of family-level taxonomy, in which five different families are identified among the analyzed genomes (marked with arrows in the internal nodes of Figure 3A). The identification of CSCGs also assisted in the construction of a global bacterial tree by introducing new protein-coding genes to a previous set of globally conserved proteins (Wu and Eisen, 2008). The global tree indicates that the *Epsilon*- and *Deltaproteobacteria* might not belong to the phylum *Proteobacteria*, but form two distinct lineages within the Bacteria (Figure 4), providing further evidence for the need to reclassify these two proteobacterial classes. The exact location of the *Epsilon*- and *Deltaproteobacteria* taxa, however, is still uncertain. While the grouping of *Epsilonproteobacteria* with *Aquificae* and the grouping of *Deltaproteobacteria* with *Acidobacteria* is in line with some studies (Wu et al., 2009; Lücker et al., 2013), others have suggested distinct branching patterns for these taxa (Rinke et al., 2013).

The non-core fraction of pan-genomes can be further divided into flexible genes that are shared among a subset of genomes, and singleton genes that are unique to individual genomes. Consistent with previous studies (Mira et al., 2010; Grote et al., 2012), our analysis of functional profiles showed that while the majority of core genes can be classified into known functional categories, the non-core genes are largely unknown (Figure 2). Moreover, the functional distribution of flexible genes suggest that the processes of signal transduction, inorganic ion transport, and energy production can be important in driving genomic variations in *Epsilonproteobacteria*. This is different from observations made on SAR11, where the processes of amino acid and carbohydrate transport dominates the flexible genes (Grote et al., 2012). In analyzing the functional profiles of flexible genes, we did not differentiate between genes that are present in the majority of the genomes from those that are present in only a few genomes, but the presence or absence of a gene in a particular genome could potentially provide useful information. This motivated us to perform a detailed study on a free-living species, *S. autotrophica*, to examine the lineage-specific versus niche-specific signals that were shared among its phylogenetic versus environmental neighbors.

The comparison of *S. autotrophica* with organisms that belong to the same genus but live in different environments, or with organisms that share the same environment but belong to different genera, has provided important insights into how the evolution of bacterial genomes is driven by either phylogenetic relatedness or environmental similarities (Figure 5). The results confirmed the presence of both lineage-specific and niche-specific signals in the flexible genome. Moreover, the data revealed multiple niche-specific gene clusters that could benefit the adaptation of *S. autotrophica* to the fluctuating and metal-rich environment of deep-sea hydrothermal vents. Detailed analyses of two niche-specific gene clusters that encode Hyf-like hydrogenase and pyruvate dehydrogenase (PYDH) complexes provided insights into the acquisition of new functions by *Epsilonproteobacteria*.

The co-occurrence of a *hyf*-like operon and a homolog of fdh-H in the genomes of vent species suggest the potential existence of a vent-specific formate hydrogenlyase complex (FHL-2). The genomic neighborhood of the *hyf*-like operon was completely unrelated (Figure 5B), suggesting that this operon was transferred independently into the vent species, potentially in response to the presence of formate as a substrate and as a way of coping with the changing environment of deep-sea hydrothermal vents. The protein phylogeny based on the E1p subunit of the PYDH complex presented distinct evolutionary paths for the type-I and type-II forms, which evolved independently in the genus *Arcobacter* and the vent species (Figure 6). The observed phylogenetic distribution and branching patterns of the two forms of PYDH may be interpreted based on two different evolutionary scenarios. In the first scenario, the type-II form was the more ancestral type that was initially present in the deepest branching lineages of *Epsilonproteobacteria*, i.e., *Nautilia* and *Nitratiruptor*, but was subsequently preserved only in the vent-inhabiting species and was lost in the other species, while the type-I form was acquired by the *Arcobacter* lineage independently via lateral gene transfer. In the second scenario, both types were absent in the common ancestor of *Epsilonproteobacteria* and were acquired by the different lineages due to adaptations that were specific to the vents (type-II form) or to the genus *Arcobacter* (type-I form). Based on the available data, the first scenario requires gene losses in multiple lineages of *Epsilonproteobacteria*. However, it is in line with the observation that the protein phylogeny of the type-II form is congruent with the species phylogeny based on both 16S rRNA and the concatenated core genes. The second scenario appears to be equally or slightly more parsimonious, but it fails to account for the observed phylogeny. At this point, it might not be possible to fully differentiate between the two scenarios due to the limited availability of genomes of free-living epsilonproteobacterial species, underlying the need for sequencing the genomes of other free-living vent and non-vent species to obtain a more comprehensive understanding of the evolution of PYDH and potentially other flexible genes of *Epsilonproteobacteria*.

Overall, the present study provided insights into the composition, flexibility, and function of epsilonproteobacterial pan-genomes and linked these factors with lineage-specific versus niche-specific gene evolution. The present study has benefited greatly from the advances in genomic sequencing and the application of such technologies to increase the breath and depth of genomic samples (Wu et al., 2009). Many of the genomes we analyzed were simply not available a few years ago. The availability of genomic data, however, is still biased toward a small number of well-studied lineages. Today we are still far from obtaining a comprehensive picture on the evolution and adaptation of bacterial genomes. This is to some extent due to the lack of cultivated strains in the under-represented branches. The development of environmental sequencing, such as metagenomics (Schmeisser et al., 2007; Johnson and Slatkin, 2009; Caro-Quintero and Konstantinidis, 2011) and single-cell genomics (Woyke et al., 2009; Marshall et al., 2012; Rinke et al., 2013), has created new frontiers in solving this problem. With more genomes of organisms from a wider range of environments going to be sequenced, we will be able to more accurately quantify the acquisition, deletion, and maintenance of genes during evolution of various bacterial genomes, hence providing improved models to simulate the process of bacterial speciation.

## Materials and methods

### Genome sequences of *Epsilonproteobacteria*

A total of 39 published genomes were collected for this study (Table S1), the majority of which came from human or animal associated species of clinical relevance, with 16 genomes representing different strains of two widely studied pathogens, *Helicobacter pylori* and *Campylobacter jejuni*. Despite this bias toward clinical species, nine complete genomes in our dataset represented free-living species from multiple genera that were isolated from a variety of environments, including deep-sea hydrothermal vents, coastal marine sediments, oil fields, and salt marshes. Additionally, we also included near-complete draft genomes from two free-living species, *Caminibacter mediatlanticus* TB-2 and *Sulfurimonas gotlandica* GD1, in order to increase the number of genomes from non-pathogenic species.

### Comparative genomics

**Figure 7:**
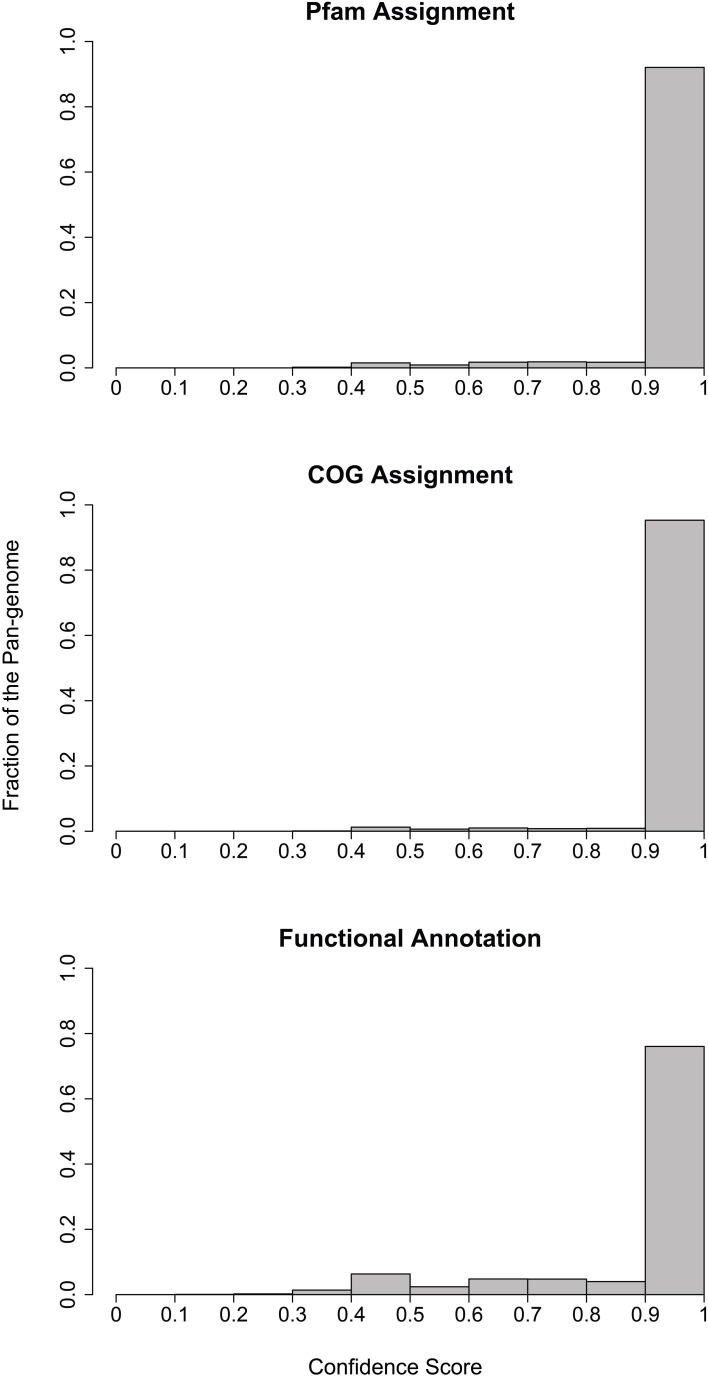
**Histogram of functional consistency within the non-redundant gene clusters.** The consistence score in the x-axis indicates the fraction of genes in a cluster that were assigned to the same function, with 1 being the most consistent and 0 being the least consistent. The y-axis indicates the fraction of multi-gene clusters in the pan-genome that carry a consistence score of a certain range.

In order to identify non-redundant gene clusters, we performed pairwise comparison on the collected genomes to identify bi-directional best hits of the encoded proteins. The determination of bi-directional best hits was based on the BLAST software (Altschul et al., 1990, 1997) using three criteria: (1) the *e*-value should be better than 0.001; (2) the alignment should cover at least 70% of the sequences; and (3) the sequence similarity should be better than 30%. Here the sequence similarity cutoff may seem low, but we used it to accommodate the orthologs in distantly related species. We showed that for the majority of the multi-gene clusters we identified, at least 90% of genes in these clusters encode proteins of the same Pfam family assignment, COG family assignment and functional annotation based on exact word matches (Figure 7). This is significant considering the many different existing ways to name identical functions. Figure 7 only presented a lower estimation of the accuracy of our approach in obtaining coherent orthologous clusters.

### Random permutation of the pan-genomes

We performed permutations on five genome sets: one inter-species, two intra-genus, as well as two intra-species sets (Table S1). At the inter-species level, we examined all of the 25 distinct species in our genome set of *Epsilonproteobacteria*; at the intra-genus level, we looked at the different species within the genera *Helicobacter* (6 species) and *Campylobacter* (6 species), disregarding strain variations over the same species by selecting one representative strain each for *H. pylori* and *C. jejuni*; at the intra-species level, we compared the different strains of *H. pylori* (10 strains) and *C. jejuni* (6 strains). All possible permutations were explored for the intra-species and intra-genus datasets. However, the inter-species dataset contains too many genomes to be fully permuted. Alternatively, we created 25,000 random permutations by randomly selecting genomes one after another over the whole dataset until all genomes has been visited.

Through each permutation, a data vector is produced to record the increased number of unique genes in the pan-genome with the step-wise addition of new genomes. Then, medians and the 25th and 75th percentile values were calculated over all permutations for each data point of the vectors. Finally, these counts were normalized by the median size of genomes, respectively (Table S2), so that pan-genomes of different datasets can be compared with one another (Figure 1).

### Power law regression model

The power law regression was performed following the approach described in Tettelin et al. (2008). The regression function *n* = σ*N*^γ^ was used to model the median sizes of the pan-genomes generated from all permutations, where *n* is the total number of non-orthologous genes in the pan-genome, *N* is the number of genomes considered, and σ and γ are free parameters (Table S2). When 0 < γ < 1, the pan-genome is considered open because it is an unbounded function over the number of genomes. When γ < 0, the pan-genome is considered closed since it approaches a constant as more genomes are considered.

### Exponential regression model

Instead of fitting the total number of genes in pan-genomes, the exponential regression model fits the number of new genes per added genome, which were implemented with an exponential decay function *F*_*s*_(*N*) = *K*_*s*_ exp[−*N*/*T*_*s*_] + *tg*(θ), where *F*_*s*_(*N*) is the number of new genes with the addition of each new genome, *tg*(θ) is the same number when *N* approaches infinity, and *K*_*s*_, *T*_*s*_, and *tg*(θ) are free parameters (Tettelin et al., 2005). Based on the estimated parameters in the exponential decay function, the pan-genomes were modeled with the formula $Pan\left(N\right)=D+\sum_{j=2}^{N}\left\{K_{s}exp\left[-j/T_{s}\right]+tg\left(\theta \right)\right\}$, where *D* is the median number of genes per sequenced genome at each dataset (Table S2).

### Phylogenomic reconstruction with concatenated core proteins

We adapted the protocol by Wu et al. for phylogenomic reconstructions (Wu and Eisen, 2008). In a first step, the individual clusters of CSCG-encoded proteins were aligned using MUSCLE (Edgar, 2004), and HMMs were built for each cluster using hmmbuild from the HMMER package (Eddy, 2011). Then, the models were used as queries to search against other genomes and the resulting alignments were trimmed adapting scripts from AMPHORA (Wu and Eisen, 2008). In a next step the trimmed alignments were concatenated with one another into a master alignment, which was further refined using Gblocks (Talavera and Castresana, 2007) to remove the less conserved columns. Finally, the refined master alignment was used as the input for PhyML (Guindon et al., 2010) for phylogenetic reconstruction.

The CSCG tree of *Epsilonproteobacteria* (Figure 3A) included six additional draft or complete genomes that were published after our initial steps of data collection. These included *Sulfurospirillum barnesii* SES-3, Uncultured *Sulfuricurvum* sp. RIFRC-1, *Arcobacter butzleri* ED-1 (Toh et al., 2011), *Arcobacter* sp. L (Toh et al., 2011), *Sulfurovum* sp. AR (Park et al., 2012), as well as the single-cell genomes of *Thiovulum* sp. ES (Marshall et al., 2012). To accommodate the incompleteness of draft genomes, we selected a subset of the CSCG-encoded proteins that occurred once in every draft genomes, and used only these as markers for tree construction. As a result, 194 of the CSCG-encoded proteins were used in the above procedure to construct the local phylogeny for *Epsilonproteobacteria*.

The global bacterial phylogeny was constructed with 37 globally conserved single copy markers (Figure 4). In addition to the 31 applied in the AMPHORA package (Wu and Eisen, 2008), we identified six additional phylogenetic markers using the HMM of core proteins: DNA gyrase subunit B (gyrB), Tryptophanyl-tRNA synthetase (TrpRS), SSU ribosomal protein S12p (S23e), LSU ribosomal protein L17p, SSU ribosomal protein S4p (S9e), and SSU ribosomal protein S15p (S13e). Among these new marker genes, GyrB (Kasai et al., 2000; Holmes et al., 2004; Peeters and Willems, 2011) and TrpRS (Rajendran et al., 2008) have been used in previous studies to determine the phylogeny of selected taxonomic groups, and the rest are ribosomal proteins.

The global bacterial tree in Figure 4 was rooted using mid-point rooting. The 16S and CSCG trees in Figure 3 were rooted based on the relative positions of different epsilonproteobacterial species at the global bacterial tree and using all other bacteria as an outgroup. As indicated with a black arrow in Figure 4, the root of *Epsilonproteobacteria* is located between *Nautiliales* and the other examined lineages.

### Protein phylogeny of PYDH

We collected representative sequences of the type-I and type-II forms of PYDH based on annotations in the UniProt database (Bairoch et al., 2005). The protein phylogeny was reconstructed with PhyML (Guindon et al., 2010) using default settings. The tree of type-I form PYDH (Figure 6A) is rooted using mid-point rooting, and the tree of type-II form PYDH (Figure 6B) is rooted with the *Eukaryotes* as an outgroup.

### Conflict of interest statement

The authors declare that the research was conducted in the absence of any commercial or financial relationships that could be construed as a potential conflict of interest.
