# Supplementary material for: Pan-genome analyses identify lineage- and niche-specific markers of evolution and adaptation in Epsilonproteobacteria
Source: Front Microbiol. 2014 Mar 19;5:110. doi: 10.3389/fmicb.2014.00110 (PMC3958643; doi:10.3389/fmicb.2014.00110)
Supplement: Supplementary file 1 [file DataSheet1.PDF]

Table S1. List of epsilonproteobacterial genomes. Genome_id, the genome ID from RefSeq database (for full genomes) or the IMG database (for draft genomes); Taxonomy ID, the taxonomy ID from NCBI; Organism Name, the full name of isolate strains. The last three columns showed the classifications of Intra-species (red background), Intra-genus (blue background), and Inter-species (green background) sets. The genera Arcobacter and Sulfurimonas were labeled but were not included in the pan-genome regression due to their small sample sizes.

Genome_id	Taxonomy ID	Organism Name	Intra-species	Intra-genus	Inter-species
NC_009850	367737	Arcobacter butzleri RM4018		Arcobacter	Epsilon
NC_014166	572480	Arcobacter nitrofigilis DSM 7299		Arcobacter	Epsilon
640963039	391592	Caminibacter mediatlanticus TB-2			Epsilon
NC_009802	360104	Campylobacter concisus 13826		Campylobacter	Epsilon
NC_009715	360105	Campylobacter curvus 525.92		Campylobacter	Epsilon
NC_008599	360106	Campylobacter fetus subsp. fetus 82-40		Campylobacter	Epsilon
NC_009714	360107	Campylobacter hominis ATCC BAA-381		Campylobacter	Epsilon
NC_003912	195099	Campylobacter jejuni RM1221	C. jejuni	Campylobacter	Epsilon
NC_009707	360109	Campylobacter jejuni subsp. doylei 269.97	C. jejuni	Campylobacter	
NC_008787	354242	Campylobacter jejuni subsp. jejuni 81-176	C. jejuni	Campylobacter	
NC_009839	407148	Campylobacter jejuni subsp. jejuni 81116	C. jejuni	Campylobacter	
NC_014802	757425	Campylobacter jejuni subsp. jejuni ICDCCJ07001	C. jejuni	Campylobacter	
NC_002163	192222	Campylobacter jejuni subsp. jejuni NCTC 11168	C. jejuni	Campylobacter	
NC_012039	306263	Campylobacter lari RM2100		Campylobacter	Epsilon
NC_008229	382638	Helicobacter acinonychis str. Sheeba		Helicobacter	Epsilon
NC_015674	1002804	Helicobacter bizzozeronii CIII-1		Helicobacter	Epsilon
NC_014810	936155	Helicobacter felis ATCC 49179		Helicobacter	Epsilon
NC_004917	235279	Helicobacter hepaticus ATCC 51449		Helicobacter	Epsilon
NC_013949	679897	Helicobacter mustelae 12198		Helicobacter	Epsilon
NC_000915	85962	Helicobacter pylori 26695	H. pylori	Helicobacter	Epsilon
NC_012973	592205	Helicobacter pylori B38	H. pylori	Helicobacter	
NC_014256	693745	Helicobacter pylori B8	H. pylori	Helicobacter	
NC_011333	563041	Helicobacter pylori G27	H. pylori	Helicobacter	
NC_008086	357544	Helicobacter pylori HPAG1	H. pylori	Helicobacter	
NC_000921	85963	Helicobacter pylori J99	H. pylori	Helicobacter	
NC_011498	570508	Helicobacter pylori P12	H. pylori	Helicobacter	
NC_014555	765963	Helicobacter pylori PeCan4	H. pylori	Helicobacter	
NC_010698	512562	Helicobacter pylori Shi470	H. pylori	Helicobacter	
NC_014560	765962	Helicobacter pylori SJM180	H. pylori	Helicobacter	
NC_012115	598659	Nautilia profundicola AmH			Epsilon
NC_014935	749222	Nitratifractor salsuginis DSM 16511			Epsilon
NC_009662	387092	Nitratiruptor sp. SB155-2			Epsilon
NC_014762	709032	Sulfuricurvum kujiense DSM 16994			Epsilon
NC_014506	563040	Sulfurimonas autotrophica DSM 16294		Sulfurimonas	Epsilon
NC_007575	326298	Sulfurimonas denitrificans DSM 1251		Sulfurimonas	Epsilon
647533121	929558	Sulfurimonas gotlandica GD1		Sulfurimonas	Epsilon
NC_013512	525898	Sulfurospirillum deleyianum DSM 6946			Epsilon
NC_009663	387093	Sulfurovum sp. NBC37-1			Epsilon
NC_005090	273121	Wolinella succinogenes DSM 1740			Epsilon

Table S2. Extrapolation of the intra-species, intra-genus, and inter-species pan-genomes using exponential vs. power law regression models. *Ks*, *Ts*, and *tg(θ)* are free parameters in the exponential decay function; *δ* and *γ* are free parameters in the power law function. The respective R-squared values indicated how well the two models fits the data at various taxa, and the Pan(30) values showed the predicted number of genes when there are 30 sequenced genomes in each dataset.

Type	Taxa	Number of Genomes	Median Genome Sizes	Exponential regression					Power law regression			
				Ks	***Ts***	***tg(θ)***	**R-squared**	**Pan(30)**	***σ***	***γ***	**R-squared**	**Pan(30)**
**intra-species**	*H. pylori*	*10*	1500	1.58E+02	6.58	62.44	0.98	4114.5	1340.98	0.29	0.99	3651
	C. jejuni	*6*	1560	5.71E+05	0.25	170.63	0.89	6734	1561.86	0.28	0.98	4093
**intra-genus**	*Helicobacter*	*6*	1587	3.47E+03	0.74	451.18	0.67	15023	1523.57	0.56	0.99	10196
	Campylobacter	*6*	1705	7.85E+02	1.98	344.69	0.93	12416	1637.38	0.51	1.00	9227
**inter-species**	*Epsilonproteobacteria*	25	1845	7.40E+02	5.35	407.71	0.98	16712	1751.01	0.66	1.00	16369

Table S3. Sulfurimonas-specific genes and their functional annotations. Annotation is the functional annotation based on the SEED database. COG_sutype, shows the functional classification based on COG family assignments. Su_aut, *Sulfurimonas autotrophica*, Su_den, *Sulfurimonas denitrificans*; Su_got, *Sulfurimonas gotlandica*; NIS, *Nitratiruptor sp*. SB155-2; SUN, *Sulfurovum sp*. NBC37-1.

Su_aut	Annotation	COG_subtype	Su_den	Su_got	NIS	SUN
307720552	Xaa-Pro aminopeptidase (EC 3.4.11.9)	Amino acid transport and metabolism	78777721	647622127	-	-
307720561	Possible acetyltransferase	Amino acid transport and metabolism	78777702	647622111	-	-
307720931	Aspartyl aminopeptidase	Amino acid transport and metabolism	78777111	647622224	-	-
307721463	Membrane alanine aminopeptidase N (EC 3.4.11.2)	Amino acid transport and metabolism	78776819	647621596	-	-
307721795	2-isopropylmalate synthase (EC 2.3.3.13)	Amino acid transport and metabolism	78778084	647621129	-	-
307719979	Alpha-ribazole-5'-phosphate phosphatase (EC 3.1.3.73)	Carbohydrate transport and metabolism	78776311	647620873	-	-
307720036	PUTATIVE INTEGRAL MEMBRANE PROTEIN	Carbohydrate transport and metabolism	78778144	647620930	-	-
307720070	Homolog of fucose/glucose/galactose permeases	Carbohydrate transport and metabolism	78778271	647620573	-	-
307720071	Aldose 1-epimerase	Carbohydrate transport and metabolism	78778270	647620572	-	-
307719962	Flagellar basal-body rod modification protein FlgD	Cell motility	78776231	647620820	-	-
307719963	Flagellar hook protein FlgE	Cell motility	78776232	647620821	-	-
307719964	Flagellar hook protein FlgE	Cell motility	78776233	647620822	-	-
307720065	Flagellar hook-associated protein FliD	Cell motility	78776403	647620578	-	-
307720466	Putative membrane protein	Cell motility	78777935	647621320	-	-
307720494	FIG00470709: hypothetical protein	Cell motility	78777801	647622186	-	-
307720638	Flagellar motor switch protein FliM	Cell motility	78776906	647622929	-	-
307720639	Flagellar motor switch protein FliN	Cell motility	78776907	647622928	-	-
307721023	Flageller protein FlgA	Cell motility	78777321	647621721	-	-
307721024	Flagellar hook-associated protein FlgL	Cell motility	78777235	647621723	-	-
307721143	methyl-accepting chemotaxis sensory transducer	Cell motility	78778126	647622112, 647623266	-	-
307721362	Flagellar biosynthesis protein FliL	Cell motility	78777038	647622378	-	-
307721502	Flagellar hook-associated protein FlgK	Cell motility	78776765	647621452	-	-
307721564	Flagellar assembly protein FliH	Cell motility	78776675	647622724	-	-
307721658	FIG00469648: hypothetical protein	Cell motility	78777877	647621351	-	-
307722075	CheC, inhibitor of MCP methylation	Cell motility	78777233	647620623	-	-
307720897, 307721292	Chemotaxis protein CheV (EC 2.7.3.-)	Cell motility	78777455	647623007	-	-
307720057	N-acetylneuraminate synthase (EC 2.5.1.56)	Cell wall/membrane/envelope biogenesis	78776794	647620579, 647622051	-	-
307720449	Transglycosylase, Slt family	Cell wall/membrane/envelope biogenesis	78777951	647621304	-	-
307720512	Membrane proteins related to metalloendopeptidases	Cell wall/membrane/envelope biogenesis	78777765	647622166	-	-
307720701	TonB-like; putative TolA function	Cell wall/membrane/envelope biogenesis	78777596	647622855	-	-
307721142	Ferric siderophore transport system, periplasmic binding protein TonB	Cell wall/membrane/envelope biogenesis	78777308	647621821	-	-
307721328	Phospholipase A1 precursor (EC 3.1.1.32, EC 3.1.1.4); Outer membrane phospholipase A	Cell wall/membrane/envelope biogenesis	78776217	647620804, 647622773	-	-
307721330	Membrane-bound lytic murein transglycosylase A precursor (EC 3.2.1.-)	Cell wall/membrane/envelope biogenesis	78777483	647622775	-	-
307721340	ABC transporter, permease protein	Cell wall/membrane/envelope biogenesis	78777514	647622241	-	-
307721483	ADP-heptose--lipooligosaccharide heptosyltransferase II (EC 2.4.1.-)	Cell wall/membrane/envelope biogenesis	78776784	647621477	-	-
307721505	FIG00469721: hypothetical protein	Cell wall/membrane/envelope biogenesis	78776762	647621449	-	-
307721682	Membrane protein related to metalloendopeptidases	Cell wall/membrane/envelope biogenesis	78776643	647622688	-	-
307721939	Membrane-bound lytic murein transglycosylase B precursor (EC 3.2.1.-)	Cell wall/membrane/envelope biogenesis	78778268	647620571	-	-
307719977	Adenosylcobinamide-phosphate guanylyltransferase (EC 2.7.7.62)	Coenzyme transport and metabolism	78776313	647620875	-	-
307719978	Cobalamin synthase	Coenzyme transport and metabolism	78776312	647620874	-	-
307719999	Nicotinate-nucleotide--dimethylbenzimidazole phosphoribosyltransferase (EC 2.4.2.21)	Coenzyme transport and metabolism	78776308	647620876	-	-
307720054	Glutamate-1-semialdehyde aminotransferase (EC 5.4.3.8)	Coenzyme transport and metabolism	78776800	647622055	-	-
307720816	NAD synthetase (EC 6.3.1.5)	Coenzyme transport and metabolism	78777049	647622363	-	-

307719991	Fe-S oxidoreductase	Energy production and conversion	78776243	647620841	-	-
307720215	oxidoreductases (related to aryl-alcohol dehydrogenases)-like	Energy production and conversion	78776530	647622534	-	-
307721531	-	Energy production and conversion	78776702	647622756	-	-
307721591	Nitroreductase	Energy production and conversion	78776719	647621414	-	-
307720001	putative lipoprotein	Function unknown	78778190	647620896	-	-
307720053	-	Function unknown	78776375	647620590	-	-
307720588	Putative lipoprotein required for motility	Function unknown	78776838	647621646	-	-
307720711	Branched-chain amino acid aminotransferase I	Function unknown	78776968	647623179	-	-
307721031	Putative periplasmic protein	Function unknown	78777243	647621730	-	-
307721056	COG1306 predicted glycoside hydrolase	Function unknown	78777266	647621770	-	-
307721150	membrane protein	Function unknown	78777315	647621838	-	-
307721201	Putative cytoplasmic protein	Function unknown	78776315	647623271	-	-
307721269	ABC transporter, putative	Function unknown	78776339	647623029	-	-
307721308	Membrane protein, putative	Function unknown	78776631, 78777567	647623286	-	-
307721455	-	Function unknown	78777698	647622099	-	-
307721456	RNA-binding S4	Function unknown	78777699	647622103	-	-
307721736	Paralysed flagella protein PflA	Function unknown	78778008	647621219	-	-
307722073	Flagellar assembly factor FliW	Function unknown	78776613	647620776, 647622652	-	-
307720642	endonuclease/exonuclease/phosphatase	General function prediction only	78776910	647621963	-	-
307720792	putative periplasmic protein	General function prediction only	78777577	647622522, 647622834	-	-
307721010	WD-40 repeat	General function prediction only	78777340	647621707	-	-
307722058	WD-40 repeat	General function prediction only	78778278	647620762	-	-
307720945, 307722009	Plasmid stabilization system	General function prediction only	78777014	647620706	-	-
307719935	Alkylphosphonate utilization operon protein PhnA	Inorganic ion transport and metabolism	78776220	647620809	-	-
307720012	Vitamin B12 ABC transporter, permease component BtuC	Inorganic ion transport and metabolism	78776316, 78777735	647620871	-	-
307720014	Haemin uptake system periplasmic haemin-binding protein	Inorganic ion transport and metabolism	78776318	647620877	-	-
307720015	TonB-dependent receptor	Inorganic ion transport and metabolism	78776319	647620878	-	-
307720906	histidine kinase	Inorganic ion transport and metabolism	78776237	647620826, 647622161	-	-
307722026	Superoxide dismutase [Fe] (EC 1.15.1.1)	Inorganic ion transport and metabolism	78777327	647621717	-	-
307720890	1-acyl-sn-glycerol-3-phosphate acyltransferase, putative	Lipid transport and metabolism	78776511	647622282	-	-
307720960	Putative 2-acylglycerophosphoethanolamine acyltransferase / acyl-acyl carrier protein synthetase (EC 6.2.1.20)	Lipid transport and metabolism	78777380	647621652	-	-
307721646	Phosphatidylserine decarboxylase-related protein	Lipid transport and metabolism	78777866	647621367	-	-
307721117	Adenylate kinase (EC 2.7.4.3)	Nucleotide transport and metabolism	78777286	647623091	-	-
307721944	CMP/dCMP deaminase, zinc-binding	Nucleotide transport and metabolism	78776348	647620620	-	-
307720222	Putative periplasmic protein	Posttranslational modification, protein turnover, chaperones	78776537	647622542	-	-
307720287	DnaJ-like protein DjlA	Posttranslational modification, protein turnover, chaperones	78776598	647622637	-	-
307720644	Serine protease precursor MucD/AlgY associated with sigma factor RpoE	Posttranslational modification, protein turnover, chaperones	78776912	647621961	-	-
307720834	OsmC-like protein	Posttranslational modification, protein turnover, chaperones	78777065	647622348	-	-
307721645	membrane protein	Posttranslational modification, protein turnover, chaperones	78777865	647621369	-	-
307721771	Heat shock protein DnaJ-like	Posttranslational modification, protein turnover, chaperones	78778036	647621195	-	-
307719959, 307720008	-	Posttranslational modification, protein turnover, chaperones	78776367	647623199	-	-
307720297	RecD-like DNA helicase YrrC	Replication, recombination and repair	78778046	647621175	-	-
307720641	Uracil-DNA glycosylase, family 1	Replication, recombination and repair	78777759	647621964	-	-

307720861	FIG00469647: hypothetical protein	Replication, recombination and repair	78777408	647623119	-	-
307720942	putative myosin heavy chain-like protein	Replication, recombination and repair	78777118	647622212	-	-
307721103	ATP-dependent RNA helicase RhlE	Replication, recombination and repair	78778139	647623272	-	-
307721105	ATP-dependent RNA helicase RhlE	Replication, recombination and repair	78777344	647621916	-	-
307721464	ATP-dependent RNA helicase RhlE	Replication, recombination and repair	78776809	647621495	-	-
307721509	Exonuclease	Replication, recombination and repair	78776758	647621445	-	-
307720866	TRAP dicarboxylate transporter, DctQ subunit, unknown substrate 6	Secondary metabolites biosynthesis, transport and catabolism	78777403	647623125	-	-
307720867	TRAP-type C4-dicarboxylate transport system, large permease component	Secondary metabolites biosynthesis, transport and catabolism	78777402	647623126	-	-
307720124	putative two-component regulator	Signal transduction mechanisms	78776409	647620561	-	-
307720495	putative signal transduction protein	Signal transduction mechanisms	78777800	647622185	-	-
307720510	FIG00469765: hypothetical protein	Signal transduction mechanisms	78777767	647622168	-	-
307720554	diguanylate cyclase (GGDEF domain) with PAS/PAC sensor	Signal transduction mechanisms	78777708	647622116	-	-
307720586	Signal-transduction regulatory protein FlgR	Signal transduction mechanisms	78776836	647621644	-	-
307720712	diguanylate cyclase (GGDEF domain) with PAS/PAC sensor	Signal transduction mechanisms	78778295	647621391	-	-
307720903	Chemotaxis regulator - transmits chemoreceptor signals to flagelllar motor components CheY	Signal transduction mechanisms	78777168	647621608	-	-
307720932	Flagellar sensory histidine kinase FlgS	Signal transduction mechanisms	78777112	647622223	-	-
307721045	response regulator receiver domain protein (CheY-like)	Signal transduction mechanisms	78777256	647621748	-	-
307721218	metal dependent phosphohydrolase	Signal transduction mechanisms	78777084	647621968, 647622302	-	-
307721317	GGDEF	Signal transduction mechanisms	78777468	647623314	-	-
307721365	response regulator receiver domain protein (CheY-like)	Signal transduction mechanisms	78777035	647620675, 647622381	-	-
307721379	GGDEF/response regulator receiver domain protein	Signal transduction mechanisms	78776937	647623214	-	-
307721685	Carbon storage regulator	Signal transduction mechanisms	78776640	647622684	-	-
307721757	diguanylate cyclase/phosphodiesterase (GGDEF & EAL domains) with PAS/PAC sensor(s)	Signal transduction mechanisms	78777555	647622822	-	-
307721904	-	Signal transduction mechanisms	78777495, 78777710	647622118	-	-
307722031	-	Signal transduction mechanisms	78778229	647620730	-	-
307722074	Chemotaxis protein CheC -- inhibitor of MCP methylation	Signal transduction mechanisms	78777232	647620624	-	-
307720005, 307720987	GGDEF FAMILY PROTEIN	Signal transduction mechanisms	78776934, 78777182	647620672, 647620880, 647622230, 647623001, 647623259	-	-
307720267, 307721219	GGDEF FAMILY PROTEIN	Signal transduction mechanisms	78776581	647621494, 647621942	-	-
307721005	RNA polymerase sigma-54 factor RpoN	Transcription	78777346	647621698	-	-
307721519	Transcription elongation factor GreA	Transcription	78776745	647621429	-	-
307721822	Response regulator	Transcription	78778118	647621537, 647621687, 647622755	-	-
307720278	Ribosomal subunit interface protein	Translation, ribosomal structure and biogenesis	78776591	647622628	-	-
307719934	-	-	78776219	647620806	-	-
307719960	pyridoxal phosphate-dependent enzyme	-	78776228	647620818	-	-
307719961	Flagellar hook-length control protein	-	78776230	647620819	-	-
307719972	-	-	78776241	647620832	-	-
307719988	-	-	78776242	647620836	-	-
307719996	-	-	78776250	647620851	-	-
307720018	Putative coiled-coil protein	-	78776325	647620882	-	-
307720023	-	-	78776334	647620887	-	-
307720031	Putative periplasmic protein	-	78778193	647620895	-	-
307720067	flagellar protein FliS	-	78776405	647620576	-	-

307720114	-	-	78776422	647620550	-	-
307720122	-	-	78776411	647620559	-	-
307720159	-	-	78776512	647622513	-	-
307720171	-	-	78776523	647622527	-	-
307720246	-	-	78776560	647622584	-	-
307720309	FIG00469824: hypothetical protein	-	78778004	647621223	-	-
307720310	Putative periplasmic protein	-	78778003	647621224	-	-
307720330	-	-	78777986	647621248	-	-
307720343	-	-	78777977	647621266	-	-
307720463	FIG00471651: hypothetical protein	-	78777938	647621317	-	-
307720465	FIG00469410: hypothetical protein	-	78777936	647621319	-	-
307720471	-	-	78778085	647622686	-	-
307720518	-	-	78777757	647622162	-	-
307720532	Putative ATP/GTP-binding protein	-	78777748	647622147	-	-
307720553	-	-	78777720	647622126	-	-
307720575	-	-	78776828	647621587	-	-
307720587	-	-	78776837	647621645	-	-
307720636	Motility integral membrane protein	-	78776904	647622931	-	-
307720645	-	-	78776913	647621960	-	-
307720715	-	-	78776882	647622289	-	-
307720743	-	-	78778168	647620962	-	-
307720750	-	-	78777781	647620946	-	-
307720824	FIG00469524: hypothetical protein	-	78777055	647622358	-	-
307720841	-	-	78777072	647622337	-	-
307720959	-	-	78777381	647621651	-	-
307720966	-	-	78776600	647621424	-	-
307720978	-	-	78777366	647621673	-	-
307720982	conserved hypothetical protein	-	78777363	647621675	-	-
307720999	-	-	78777647	647622821	-	-
307721067	FIG00469406: hypothetical protein	-	78777276	647621780	-	-
307721121	-	-	78777289	647621798	-	-
307721131	FIG00469478: hypothetical protein	-	78777297	647621809	-	-
307721213	-	-	78777190	647621934	-	-
307721296	-	-	78777460	647622994	-	-
307721298	-	-	78777461	647622991	-	-
307721300	-	-	78777464	647622989	-	-
307721384	-	-	78776933	647623224	-	-
307721401	-	-	78777666	647621995	-	-
307721454	-	-	78777697	647622098	-	-
307721480	-	-	78776786	647621480	-	-
307721484	FIG00760460: hypothetical protein	-	78776782	647621474	-	-
307721503	FIG00469453: hypothetical protein	-	78776764	647621451	-	-
307721504	FlgM protein	-	78776763	647621450	-	-
307721508	Dihydroneopterin aldolase (EC 4.1.2.25)	-	78776759	647621446	-	-
307721510	-	-	78776757	647621444	-	-
307721528	PUTATIVE OUTER MEMBRANE PROTEIN	-	78777839	647623275	-	-
307721557	-	-	78776682	647622730	-	-
307721580	-	-	78776739	647621422	-	-
307721596	Putative lipoprotein	-	78776715	647621409	-	-
307721604	-	-	78776710	647621407	-	-
307721607	-	-	78776708	647621404	-	-
307721608	-	-	78776707	647621403	-	-
307721609	-	-	78776706	647621402	-	-
307721634	-	-	78777860	647621382	-	-
307721636	-	-	78777861	647621381	-	-
307721723	FIG00469551: hypothetical protein	-	78778056	647621172	-	-
307721726	FIG00469729: hypothetical protein	-	78778059	647621169	-	-
307721730	-	-	78778062	647621162	-	-
307721774	membrane protein	-	78778067	647621157	-	-
307721775	membrane protein	-	78778068	647621156	-	-
307721829	membrane protein	-	78778101	647621084	-	-
307721851	-	-	78778130	647621062	-	-
307721884	FIG00469556: hypothetical protein	-	78776277	647621036	-	-
307721931	-	-	78776406	647620564	-	-
307722021	FIG00469742: hypothetical protein	-	78778221	647620715	-	-
307722043	-	-	78778245	647620742, 647620743	-	-
307722072	-	-	78778291	647620775	-	-

Table S4. Vent-specific genes and their functional annotations. Annotation is the functional annotation based on the SEED database. COG_sutype, shows the functional classification based on COG family assignments. Su_aut, Sulfurimonas autotrophica, Su_den, Sulfurimonas denitrificans; Su_got, Sulfurimonas gotlandica; NIS, Nitratiruptor sp. SB155-2; SUN, Sulfurovum sp. NBC37-1.

Su_aut	Annotation	COG_subtype	Su_den	Su_got	NIS	SUN
307720819	amino acid transporter	Amino acid transport and metabolism	-	-	152990537, 152991485	152992623, 152992627
307720409	Phosphoglycerate mutase (EC 5.4.2.1)	Carbohydrate transport and metabolism	-	-	152990666	152992879
307720399	Glycogen synthase, ADP-glucose transglucosylase (EC 2.4.1.21)	Carbohydrate transport and metabolism	-	-	152990683	152992858
307720388	Glycogen phosphorylase (EC 2.4.1.1)	Carbohydrate transport and metabolism	-	-	152990689, 152990692	152992867
307720400	1,4-alpha-glucan (glycogen) branching enzyme, GH-13-type (EC 2.4.1.18)	Carbohydrate transport and metabolism	-	-	152990682	152992857
307720384	Fructose-bisphosphate aldolase, archaeal class I (EC 4.1.2.13)	Carbohydrate transport and metabolism	-	-	152990673	152992802
307720395	Alpha-amylase (EC 3.2.1.1)	Carbohydrate transport and metabolism	-	-	152990686	152992862
307720394	glycoside hydrolase family 57	Carbohydrate transport and metabolism	-	-	152990687	152992863
307720392	Phosphoglucomutase (EC 5.4.2.2)	Carbohydrate transport and metabolism	-	-	152990691	152992866
307720359	outer membrane efflux protein, putative	Cell wall/membrane/envelope biogenesis	-	-	152989826	152993015
307720360	Putative membrane fusion component of efflux system	Cell wall/membrane/envelope biogenesis	-	-	152989827	152993014
307721661	-	Cell wall/membrane/envelope biogenesis	-	-	152991539	152993957
307720408	Lipoate synthase	Coenzyme transport and metabolism	-	-	152990671	152992558
307721236	ABC transport system, permease component YbhR	Defense mechanisms	-	-	152991467	152991876
307720361	RND multidrug efflux transporter; Acriflavin resistance protein	Defense mechanisms	-	-	152989828	152993013
307721235	Probable permease of ABC transporter	Defense mechanisms	-	-	152991468	152991877
307721238	secretion protein HlyD family protein	Defense mechanisms	-	-	152991464	152991873
307719950	Cadmium efflux system accessory protein	Transcription	-	-	152990654	152992380
307720358	transcriptional regulator, TetR family	Transcription	-	-	152989825	152993016
307720398	ROK family Glucokinase with ambiguous substrate specificity	Transcription	-	-	152990684	152992859
307719973	Trk system potassium uptake protein TrkA	Inorganic ion transport and metabolism	-	-	152991480	152991843
307720426	Ferrous iron transport protein B	Inorganic ion transport and metabolism	-	-	152989847	152992974
307720161	NnrS protein involved in response to NO	Inorganic ion transport and metabolism	-	-	152989874	152992825
307721232	major facilitator superfamily MFS_1	Inorganic ion transport and metabolism	-	-	152991465	152991874
307719974	Potassium uptake protein TrkH	Inorganic ion transport and metabolism	-	-	152991481	152991844
307720994	Potassium channel protein	Inorganic ion transport and metabolism	-	-	152991458	152993184
307720406	Dihydrolipoamide acetyltransferase component of pyruvate dehydrogenase complex (EC 2.3.1.12)	Energy production and conversion	-	-	152990678	152992800
307720407	Dihydrolipoamide dehydrogenase of branched-chain alpha-keto acid dehydrogenase (EC 1.8.1.4)	Energy production and conversion	-	-	152990677	152992799
307720404	Pyruvate dehydrogenase E1 component alpha subunit (EC 1.2.4.1)	Energy production and conversion	-	-	152990680	152992563
307720405	Pyruvate dehydrogenase E1 component beta subunit (EC 1.2.4.1)	Energy production and conversion	-	-	152990679	152992564
307721952	Formate hydrogenlyase subunit 7	Energy production and conversion	-	-	152990463	152991860
307721953	Hydrogenase-4 component G (EC 1.-.-.-)	Energy production and conversion	-	-	152990464	152991859
307721954	Formate hydrogenlyase subunit 3/Multisubunit Na+/H+ antiporter, MnhD subunit	Energy production and conversion	-	-	152990465	152991858

307721955	Hydrogenase-4 component E (EC 1.-.-.-)	Energy production and conversion	-	-	152990466	152991857
307721956	Formate hydrogenlyase subunit 4	Energy production and conversion	-	-	152990467	152991856
307721957, 307721958	Hydrogenase-4 component B (EC 1.-.-.-) / Formate hydrogenlyase subunit 3	Energy production and conversion	-	-	152990468	152991855
307720396	Galactose-1-phosphate uridylyltransferase (EC 2.7.7.10)	Energy production and conversion	-	-	152990685	152992861
307720125	NADP-dependent malic enzyme (EC 1.1.1.40)	Energy production and conversion	-	-	152991381	152992111
307720041	Peptide methionine sulfoxide reductase MsrA (EC 1.8.4.11)	Posttranslational modification, protein turnover, chaperones	-	-	152990862	152992273
307720597	Putative protein-S-isoprenylcysteine methyltransferase	Posttranslational modification, protein turnover, chaperones	-	-	152990422	152992901
307721612	Peptide methionine sulfoxide reductase MsrB (EC 1.8.4.12)	Posttranslational modification, protein turnover, chaperones	-	-	152989803	152993277
307721950	A/G-specific adenine glycosylase (EC 3.2.2.-)	Replication, recombination and repair	-	-	152989955	152991607
307721200	G:T/U mismatch-specific uracil/thymine DNA-glycosylase	Replication, recombination and repair	-	-	152990863	152992204
307722024	Resolvase/integrase	Replication, recombination and repair	-	-	152989893	152991667
307721112	C4-type zinc finger protein, DksA/TraR family	Signal transduction mechanisms	-	-	152990821	152993038
307720225	-	Signal transduction mechanisms	-	-	152991401	152992643
307720827	Prolyl-tRNA synthetase (EC 6.1.1.15), bacterial type	Translation, ribosomal structure and biogenesis	-	-	152990768	152992232
307721157	2'-5' RNA ligase	Translation, ribosomal structure and biogenesis	-	-	152991336	152991756
307720131	-	Function unknown	-	-	152990695	152992510
307720422	-	Function unknown	-	-	152989829	152993011
307720429	-	Function unknown	-	-	152989956	152992626
307720815	COG2833: uncharacterized protein	Function unknown	-	-	152990790	152993216
307721104	MgtC family	Function unknown	-	-	152990675	152992875
307720383, 307720820	-	Function unknown	-	-	152989767	152992871
307720402	-	General function prediction only	-	-	152989841	152992976
307721237	putative ABC transporter ATP-binding protein	General function prediction only	-	-	152991466	152991875
307721276	Decarboxylase family protein	General function prediction only	-	-	152990674	152992798
307720419, 307720596	-	General function prediction only	-	-	152989835	152991809
307720362	-	-	-	-	152989845	152992992
307720380	Probable transmembrane protein	-	-	-	152991148	152992625
307720403	-	-	-	-	152989840	152992977
307720526	-	-	-	-	152990305	152992785
307720672	-	-	-	-	152990983	152993369
307720821	-	-	-	-	152991514	152992624
307721461	-	-	-	-	152990074	152991787
307721733	-	-	-	-	152991162	152993859
307721975	-	-	-	-	152991482	152992286

Table S5. List of proteins in the pyruvate dehydrogenase complex (PYDH). The *Arcobacter* carry the type-I form of E1p component, which contains a single subunit (blue). The vent species carry the type-II form of E1p component that contains two subunits, alpha and beta (red).

**Function**	Arcobacter butzleri RM4018	Arcobacter nitrofigilis DSM 7299	Nautilia profundicola AmH	Nitratifractor salsuginis DSM 16511	Nitratiruptor sp. SB155-2	Sulfurimonas autotrophica DSM 16294	Sulfurovum sp. NBC37-1
Pyruvate dehydrogenase E1 component alpha subunit	-	-	224372803	319956741	152990680	307720404	152992563
Pyruvate dehydrogenase E1 component beta subunit	-	-	224372802	319956742	152990679	307720405	152992564
Dihydrolipoamide acetyltransferase (E2p)	157737714	296274129	224372801	319956743	152990678	307720406	152992800, 152992561
Dihydrolipoamide dehydrogenase (LPD)	-	296273524	224372800	319956745	152990677	307720407	152992799
Pyruvate dehydrogenase E1 component	157737713	296274128	-	-	-	-	-
